# Broader Epstein–Barr virus–specific T cell receptor repertoire in patients with multiple sclerosis

**DOI:** 10.1084/jem.20220650

**Published:** 2022-09-01

**Authors:** Tilman Schneider-Hohendorf, Lisa Ann Gerdes, Béatrice Pignolet, Rachel Gittelman, Patrick Ostkamp, Florian Rubelt, Catarina Raposo, Björn Tackenberg, Marianne Riepenhausen, Claudia Janoschka, Christian Wünsch, Florence Bucciarelli, Andrea Flierl-Hecht, Eduardo Beltrán, Tania Kümpfel, Katja Anslinger, Catharina C. Gross, Heidi Chapman, Ian Kaplan, David Brassat, Hartmut Wekerle, Martin Kerschensteiner, Luisa Klotz, Jan D. Lünemann, Reinhard Hohlfeld, Roland Liblau, Heinz Wiendl, Nicholas Schwab

**Affiliations:** 1 Department of Neurology with Institute of Translational Neurology, University of Münster, Münster, Germany; 2 Institute of Clinical Neuroimmunology, University Hospital and Biomedical Center, Ludwig-Maximilians Universität München, Munich, Germany; 3 Biomedical Center, Faculty of Medicine, Ludwig-Maximilians Universität München, Martinsried, Germany; 4 Munich Cluster of Systems Neurology (SyNergy), Munich, Germany; 5 Toulouse Institute for infectious and inflammatory diseases (Infinity), University of Toulouse, Centre National de la Recherche Scientifique, Institut National de la Santé et de la Recherche Médicale, Université Paul Sabatier, Toulouse, France; 6 Adaptive Biotechnologies, Seattle, WA; 7 Roche Sequencing Solutions, Pleasanton, CA; 8 F. Hoffmann-La Roche Ltd, Basel, Switzerland; 9 Philipps-University, Department of Neurology, Marburg, Germany; 10 Institute of Legal Medicine, Ludwig-Maximilians Universität München, Munich, Germany; 11 Institute for Biological Intelligence, Martinsried, Germany

## Abstract

Epstein–Barr virus (EBV) infection precedes multiple sclerosis (MS) pathology and cross-reactive antibodies might link EBV infection to CNS autoimmunity. As an altered anti-EBV T cell reaction was suggested in MS, we queried peripheral blood T cell receptor β chain (TCRβ) repertoires of 1,395 MS patients, 887 controls, and 35 monozygotic, MS-discordant twin pairs for multimer-confirmed, viral antigen–specific TCRβ sequences. We detected more MHC-I–restricted EBV-specific TCRβ sequences in MS patients. Differences in genetics or upbringing could be excluded by validation in monozygotic twin pairs discordant for MS. Anti–VLA-4 treatment amplified this observation, while interferon β– or anti-CD20 treatment did not modulate EBV-specific T cell occurrence. In healthy individuals, EBV-specific CD8^+^ T cells were of an effector-memory phenotype in peripheral blood and cerebrospinal fluid. In MS patients, cerebrospinal fluid also contained EBV-specific central-memory CD8^+^ T cells, suggesting recent priming. Therefore, MS is not only preceded by EBV infection, but also associated with broader EBV-specific TCR repertoires, consistent with an ongoing anti-EBV immune reaction in MS.

## Teaser

The EBV-specific T cell receptor repertoire is broader in multiple sclerosis patients, even in diseased siblings of discordant monozygotic twin pairs, indicating an ongoing immune response to Epstein–Barr virus in multiple sclerosis patients, which might hold clues for multiple sclerosis pathogenesis and future therapeutic or preventive avenues.

## Introduction

EBV seroconversion has been shown in large epidemiological studies to precede clinical signs of multiple sclerosis (MS; Bjornevik et al., 2022; Levin et al., 2010), confirming that EBV infection is necessary but not sufficient for disease initiation and associated central nervous system (CNS) damage. Additionally, antibody cross-reactivity was detected between a latent viral epitope of Epstein-Barr nuclear antigen-1 (EBNA-1) and a CNS autoantigen (GlialCAM) in a subset of patients as a humoral component of—and potential link to—MS pathology (Aloisi and Salvetti, 2022; Lanz et al., 2022). While relapsing-remitting MS (RRMS) is specifically characterized by the presence of B- and plasma cells in the cerebrospinal fluid (CSF; Gross et al., 2021), T cells and macrophages dominate CNS immune cell infiltrates in MS (Kuhlmann et al., 2008) and relapses are associated with influx of T cells (Schneider-Hohendorf et al., 2021). This hints at recurrent antigen drainage from the CNS into the periphery and subsequent recruitment of peripheral cytotoxic as well as T helper cells (Bar-Or et al., 2021). It has been suggested previously that peripheral T cells show increased cytokine response to latent EBNA-1 epitopes (Lunemann et al., 2006) with presumed cross-reactivity to myelin (Lunemann et al., 2008). However, it has also been discussed that the anti-EBV T cell response in MS patients targets lytic components, indicating ongoing EBV activity (Angelini et al., 2013; Lassmann et al., 2011) and/or insufficient EBV control (Cencioni et al., 2017; Pender et al., 2009).

## Results and discussion

### Quantification of EBV-specific, MHC-I–restricted TCRβ sequences in HLA-A*02–positive MS patients and healthy controls

**Figure 1.**
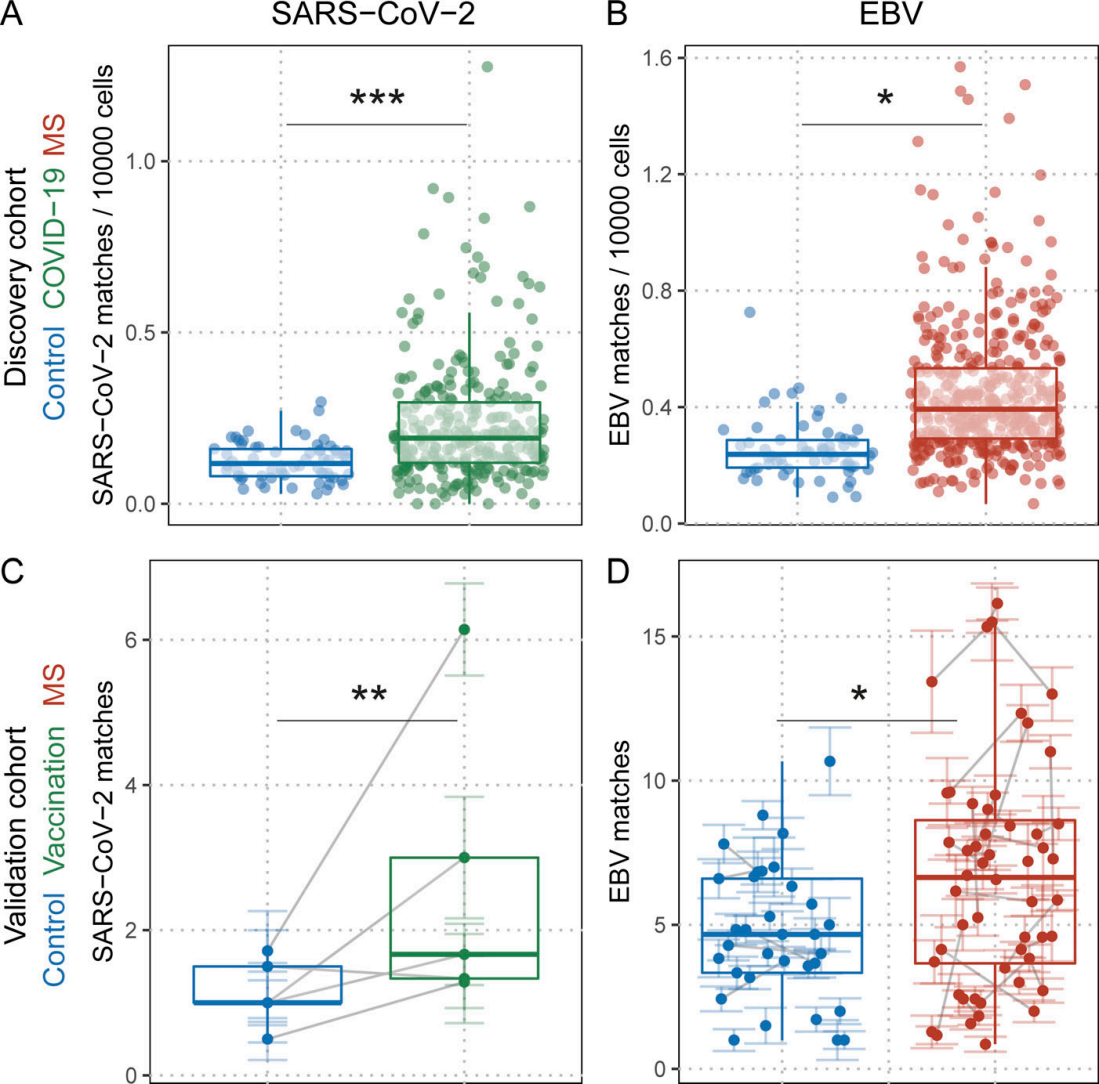
**Quantification of SARS-CoV-2– and EBV-specific T cell rearrangements in TCRβ repertoires of the discovery cohort and the validation cohort. (A and B)** SARS-CoV-2 (q_COVID-19_ = 4e^−05^; n_HD_ = 62; n_COVID-19_ = 278; A) and EBV (q_MS_ = 0.01088; n_HD_ = 62; n_MS_ = 430; B) TCRβ sequence matches quantified in HD (blue dots), patients with acute COVID-19 (COVID-19, green dots), and MS patients (red dots); *q* values indicate adjusted significance of disease state (COVID-19 or MS) in linear models with the covariates sequencing depth, age, sex, and HLA. **(C)** SARS-CoV-2 TCRβ sequence matches quantified in HD before their first (blue dots) and after their second SARS-CoV-2 vaccination (green dots; q_Vaccination_ = 0.00196; *n* = 5). Colored lines indicate standard error of the mean of the biological replicates (sequencing pools) for the respective sample, and gray lines connect samples from the same individual. *q* values indicate adjusted significance of vaccination in linear mixed models with the covariates sequencing depth, vaccination status, and sequencing pools nested within samples within individuals. **(D)** EBV TCRβ sequence matches quantified in control donors (blue dots), and MS patients (red dots; q_MS_ = 0.0298172; n_Control_ = 27; n_MS_ = 25). Colored lines indicate standard error of the mean of the sequencing pools for the respective sample, and gray lines connect samples from the same individual. *q* values indicate adjusted significance of MS in linear mixed models with the covariates sequencing depth, age, sex, treatment, and sequencing pools nested within samples within individuals.

**Figure S1.**
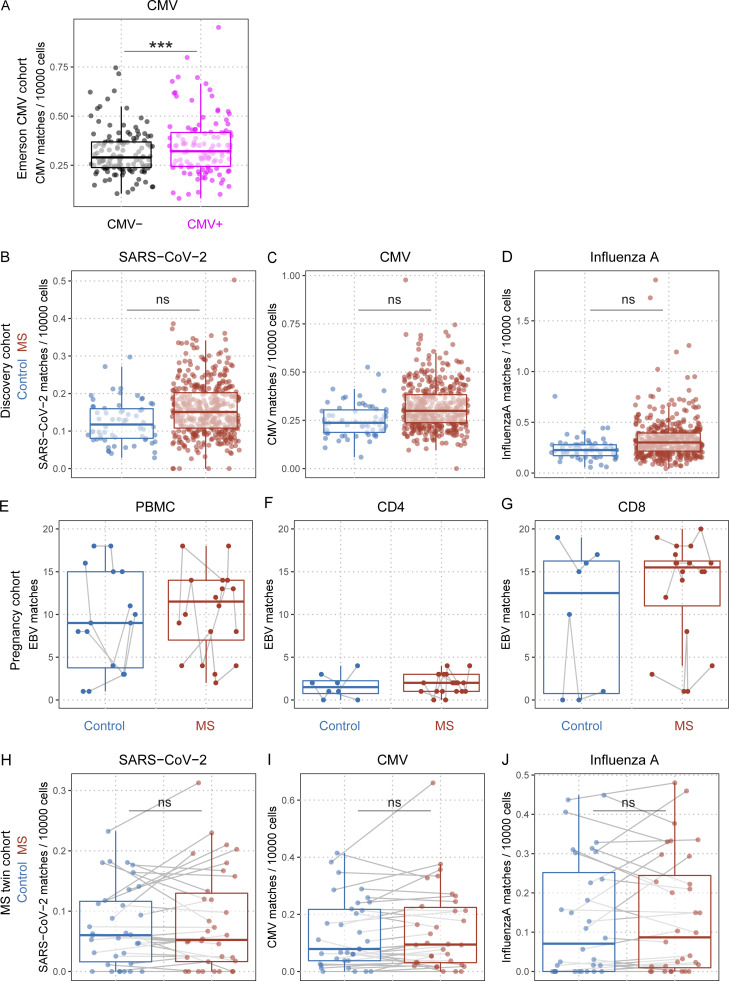
**Quantification of pathogen-specific TCRβ sequences in TCRβ repertoires. (A)** CMV TCRβ sequence matches quantified in CMV-seronegative HD (black dots) and CMV-seropositive HD (magenta dots; Emerson et al., 2017) (p_CMV Serostatus_ = 3e^−07^; n_CMV−_ = 129; n_CMV+_ = 115); P value indicates significance of serostatus in linear models with the covariates sequencing depth, age, sex, and HLA. **(B–D)** SARS-CoV-2 (B), CMV (C), and influenza A (D). TCRβ sequence matches quantified in HD (blue dots) and MS patients (red dots; q_MS_ = 1; n_Control_ = 62; n_MS_ = 430); *q* values indicate adjusted significance of disease state (MS) in linear models with the covariates sequencing depth, age, sex, and HLA. **(E–G)** EBV-specific TCRβ sequence matches quantified in pregnant HD (blue dots and boxes) and pregnant MS patients (red dots and boxes; p_MS_ = 0.0001047, n_Control_ = 4, n_MS_ = 4; p_CD4_ = 2e^−06^, n_Control_ = 2, n_MS_ = 4; p_CD8_ = 0.0093175, n_Control_ = 2, n_MS_ = 4); gray lines connect samples from the same individual; P values indicate adjusted significance of MS (MS mean higher than HD mean), CD4 (CD4 mean lower than PBMC mean), and CD8 (CD8 mean higher than PBMC mean), in a combined linear mixed model with the covariates sequencing depth, age, pregnancy phase, cell subset, and HLA. **(H–J)** SARS-CoV-2 (H), CMV (I), and influenza A (J). TCRβ sequence matches quantified in monozygotic twins discordant for MS (healthy twin siblings, blue dots; and MS twin siblings, red dots; q_MS_ = 1; n_Control_ = 35; n_MS_ = 35); gray lines indicate twinship; *q* values indicate adjusted significance of disease state (MS) in a linear mixed model with the covariates sequencing depth, age, disease status (HD, MS), symptomatic EBV infection in childhood, HLA, and twinship.

In light of the finding that EBV infection precedes the development of MS and that some MS patients showed cross-reactive antibody binding to EBV, as well as CNS structures, we evaluated the hypothesis, if the TCR repertoire against EBV might also be altered in MS patients. For this, we collected multimer-confirmed TCRβ sequences from public, curated databases of peer-reviewed studies, resulting in 528 EBV-specific, 840 CMV-specific, 381 influenza A virus–specific and 644 SARS-CoV-2–specific TCRβ sequences together with their MHC restriction elements (detailed in Table S1). These sequences were then queried and quantified in deep TCRβ repertoires (ImmunoSEQ Assay) from peripheral blood of HLA-A*02–positive MS patients and controls, only assessing database sequences matched to the HLA combination of the respective individual (Table 1). To test the feasibility of the analysis approach, we first queried 62 healthy controls and a published dataset of 278 COVID-19 patients (Snyder et al., 2020) for SARS-CoV-2–specific TCRβ sequences, and found that COVID-19 patients presented with significantly more matches in their repertoires (Fig. 1 A; q = 4 × 10^−5^). Additionally, a previously published cohort of individuals with positive CMV serostatus (Emerson et al., 2017) presented with more CMV-specific sequences than individuals with negative CMV serostatus (Fig. S1 A). All obtained EBV-specific TCRβ sequences were MHC-I restricted and consisted of 404 sequences against lytic and 124 against latent epitopes (Table S1). Of note, <5% of the extracted database EBV sequences were specific for latent EBNA-1–derived epitopes. To cross-validate the specificity of these sequences for an individual’s HLA type, the sequences were compared with previously published, highly specific HLA-classifying patterns (DeWitt et al., 2018), which showed that 57 of the 528 EBV-specific sequences could be found in HLA classifiers, 56 of them (98%) in the HLA for which the original multimer staining was specific (Table S2). This suggests that (a) the database clones can be detected with high specificity in individuals expressing the respective HLA type and (b) HLA classifier patterns contain sequences specific for EBV, which could be expected given that EBV is such a highly prevalent pathogen. Assessment of the HLA-A*02–positive discovery cohort of 430 MS patients and 62 healthy controls revealed that MS patients’ TCRβ repertoires contained a higher number of TCR sequences matching with EBV-specific database entries (Fig. 1 B; q = 1.1 × 10^−2^; Table S1). To assess the specificity of this finding for EBV, we also queried database-derived TCR sequences specific for SARS-CoV-2, CMV, and influenza A virus. However, no differences were observed for MS patients compared to healthy controls (Fig. S1, B–D). Of note, the adjusted effect size of the EBV matches in MS patients was +2.2 and, therefore, comparable to the +2.9 in COVID-19 patients with regard to SARS-CoV-2 matches, which is surprisingly high considering the acute antiviral response in previously unexposed COVID-19 patients and that, due to their age, almost all individuals from the discovery cohort can safely be assumed to be EBV seropositive (Abrahamyan et al., 2020).

**Table 1.** Cohorts and sequencing characteristics

Parameter	Discovery cohort			MS twin cohort		Validation cohort	
Name	COVID-19	HD	MS	HD	MS	Control	MS
Data source	Data from Snyder et al. (2020)	Previously unpublished data					
Assay	immunoSEQ					immunoPETE	
Number of individuals	607	229	1,336	35	35	51	59
HLA	Imputed	Typed	Typed	Typed	Typed	Imputed	Imputed
Sequencing depth	391,829 (182,941)	564,815 (148,717)	380,234 (171,527)	670,874 (183,708)	616,687 (209,577)	42,759 (14,585)	41,737 (12,769)
	[Productive templates per sample]					[Input α β T cells per sequencing pool]	
Age (yr)	61 (18)	52 (17)	37 (9)	40 (11)	40 (11)	45 (14)	41 (10)
Sex (female)	54.1%	51.1%	75.7%	80.0%	80.0%	52.4%	64.6%
Details of the HLA-A*02–positive subcohort							
Number of HLA-A*02–positive individuals	278	62	430			27	25
Anti–VLA-4: untreated/treated			248/73		33/2		17/8
IFNβ: treatment-naive/treated			29/123		10/10		
Anti-CD20: before/after treatment					34/1		14/14
Vaccination: before/after						5/5	

Given values are mean (SD) for scalar variables and *n* (%) for categorical variables.

With all of the queried EBV-specific TCR sequences being MHC class-I restricted, the results should pertain to CD8^+^ T cells. This was clearly confirmed by assessing TCRβ repertoire data from an additional, published cohort of MS patients and controls with available separation of PBMC, CD4^+^, and CD8^+^ T cells (Ramien et al., 2019; Fig. S1, E–G). This complements the knowledge of EBNA1-specific responses in MS, which some studies suggest are mostly CD4^+^ T cells (Lunemann et al., 2006), because this study addresses involvement of EBV-specific CD8^+^ T cells, which have, as tissue-resident memory cells, been shown to contribute to CNS pathology (Chang et al., 2014; Friese and Fugger, 2009; Hausler et al., 2021).

### Independent validation of the broader EBV-specific TCRβ repertoire in MS patients

**Figure S2.**
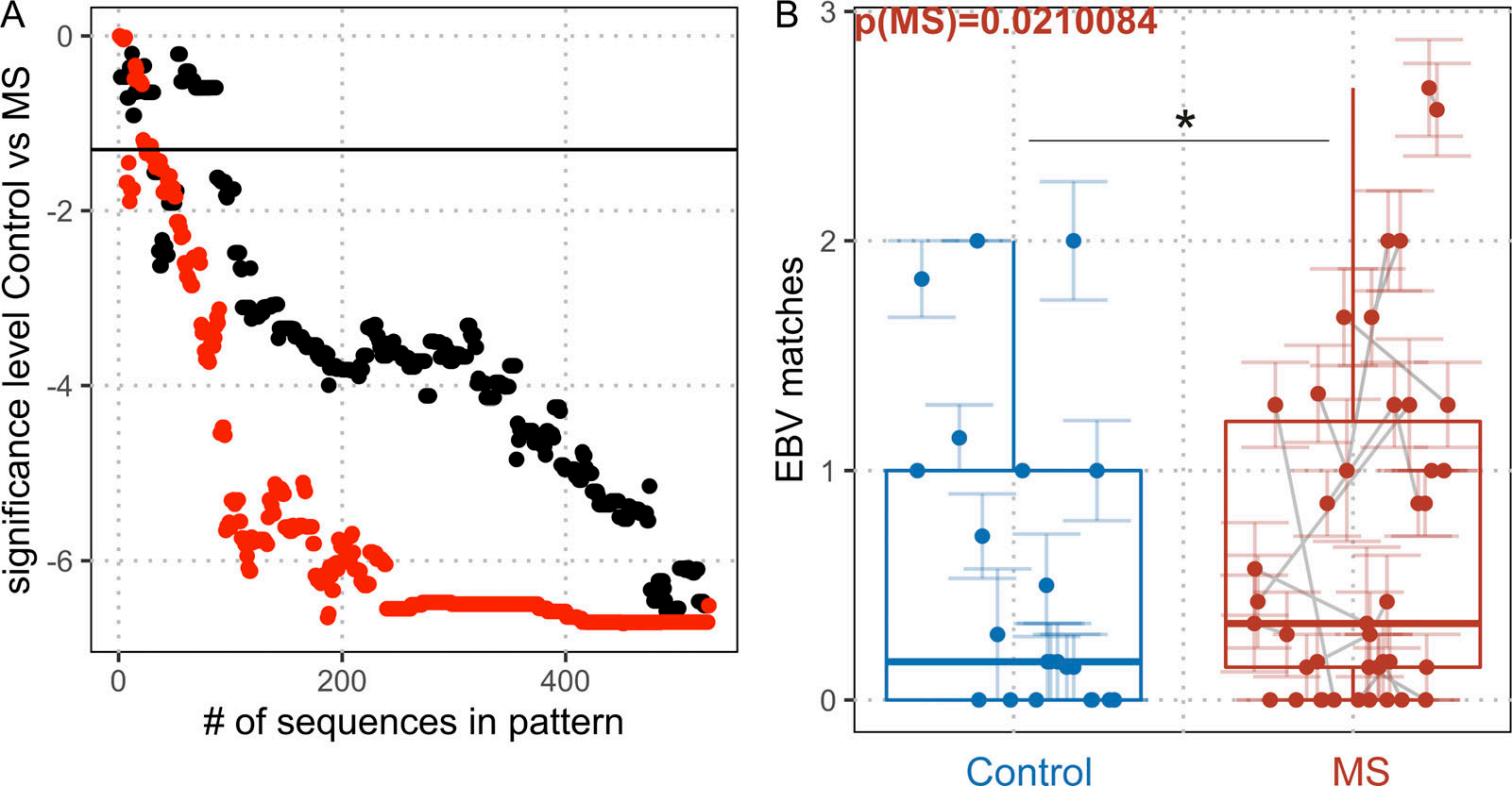
**MS association of EBV-specific TCR sequences. (A)** Shown is log_10_ of the P value (control versus MS) within the validation cohort when quantifying matches of 1–528 EBV-specific sequences. The sequences were added incrementally either randomly (black dots) or according to their MS association determined within the discovery cohort using lasso regression models (red dots, MS as target variable, adjustment for HLA background). The black line is drawn at log_10_ of 0.05 as the level of significance. **(B)** The top seven MS-associated EBV TCRβ sequences matched in control donors (blue dots) and MS patients (red dots) of the validation cohort; p_MS_ = 0.0210084; n_Control_ = 27; n_MS_ = 25). Colored lines indicate standard error of the mean of the sequencing pools for the respective sample; gray lines connect samples from the same individual. P value indicates adjusted significance of MS in linear mixed models with the covariates sequencing depth, age, sex, treatment, and sequencing pools nested within samples within individuals.

In order to confirm the broader EBV-specific TCRβ repertoire in MS patients, an independent validation cohort was sequenced, using a different immunosequencing method (immunoPETE assay). This cohort contained samples of (a) seven healthy donors (HD), five of them longitudinally sampled before the first and 6 wk after the second SARS-CoV-2 mRNA vaccination; (b) 17 MS patients before, and 6 and 12 mo after anti-CD20 therapy (ocrelizumab); as well as (c) eight MS patients at two time points 6 mo apart during therapy with a VLA-4–blocking antibody (natalizumab). Additionally, it contained (d) 20 samples from patients with autoimmune encephalitis, added in as neuroinflammatory non-MS controls, to further investigate MS specificity. This assessment validated our previous results, i.e., detection of SARS-CoV-2 matches after vaccination as a positive control (Fig. 1 C; q = 2 × 10^−3^) and the broader EBV-specific TCR repertoire in MS patients (Fig. 1 D; q = 3 × 10^−2^). Of note, the adjusted effect size was +2.1 EBV-specific matches in the MS patients compared to controls and, therefore, highly comparable to the discovery cohort. To evaluate, whether some of the database TCRβ sequences might not only be specific for EBV, but also to a certain degree associated with MS, the 528 sequences were subjected to lasso regression models within the HLA-A*02 discovery cohort and the MS condition as target variable, adjusting for the individuals’ HLA type. The sequences were then ranked according to their P value with regard to prevalence in MS. Compared with a random shuffling of the sequences, incremental inclusion of MS-ranked sequences into a pattern reached the optimal P value in the validation cohort much quicker (Fig. S2 A) and a pattern of the top seven ranked MS-associated EBV sequences from the discovery cohort was sufficient to reach a significant difference comparing controls versus MS patients when quantifying them in the validation cohort (Fig. S2 B; ranks detailed in Table S1). This suggests that MS patients share a higher prevalence of EBV-specific TCRs, which is generalizable between MS cohorts, and differs significantly from controls. However, EBV-specific TCR sequences alone are not sufficient to classify MS patients and discriminate them from controls in form of a biomarker.

### Evaluation of the EBV-specific TCRβ repertoire in monozygotic twin pairs discordant for MS

**Figure 2.**
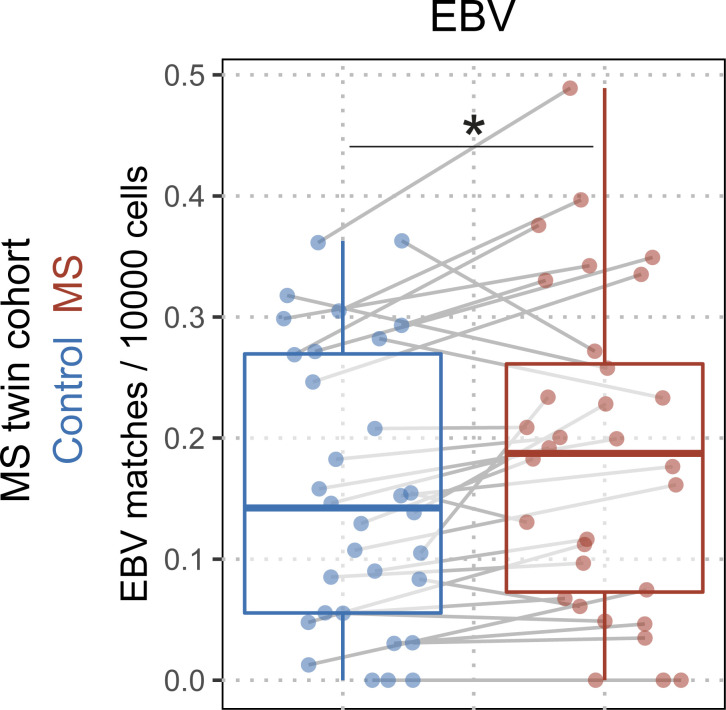
**Higher number of EBV matches in the TCRβ repertoires of the siblings with MS in monozygotic twins discordant for MS.** EBV-specific TCRβ sequence matches quantified in syngeneic twins discordant for MS (healthy twin siblings, blue dots; and MS twin siblings, red dots; q_MS_ = 0.02868; n_Control_ = 35; n_MS_ = 35); gray lines indicate twinship, *q* values indicate adjusted significance of disease state (MS) in a linear mixed model with the covariates sequencing depth, age, disease status (HD, MS), symptomatic EBV infection in childhood, HLA, and twinship.

To evaluate whether the increased sequence matches specific to EBV were simply related to underlying MS-associated genetics or early environmental differences, or rather to the disease itself, we sequenced and analyzed 35 monozygotic twin pairs discordant for MS. Despite matching positive EBV serostatus, this comparison revealed that only EBV-specific (Fig. 2; q = 2.9 × 10^−2^), but not SARS-CoV-2–, CMV-, or influenza A–specific, TCRβ sequences (Fig. S1, H–J) showed a higher number of matches in the MS twin sibling compared to their healthy sibling. Again, the adjusted effect size was comparable to the discovery and the validation cohort. The information about whether any of the siblings had a symptomatic EBV infection in childhood/infectious mononucleosis was taken into account in the modeling, but did not influence the result. Together, these data confirm elevated numbers of EBV-specific clonotypes in peripheral T cells in MS and suggest its independence from known genetic and early environmental factors.

### Assessment of EBV epitope–specific differences in MS patients and controls

To assess individual EBV epitopes in a complementary approach, subanalyses were conducted using the whole dataset (HLA-A*02–negative and –positive individuals; Table 1), grouping only donors and database TCRβ sequences fitting to the restriction element of an individual epitope in each subanalysis (Table S3). Within these 10 subanalyses, 2 lytic (RAKFKQLL in HLA-B*08 and EPLPQGQLTAY in HLA-B*35, both of BZLF-1) and 3 latent (HPVGEADYFEY [EBNA-1 in HLA-B*35], RPPIFIRRL [EBNA-3 in HLA-B*07], and FLRGRAYGL [EBNA-3 in HLA-B*08]) epitopes reached significance in the discovery cohort (detailed in Table S3). The biggest effect size of +1.1 was detected for the RAKFKQLL epitope of the BZLF-1 protein in HLA-B*08.

### Influence of MS treatments on peripheral EBV-specific TCRβ sequences

**Figure 3.**
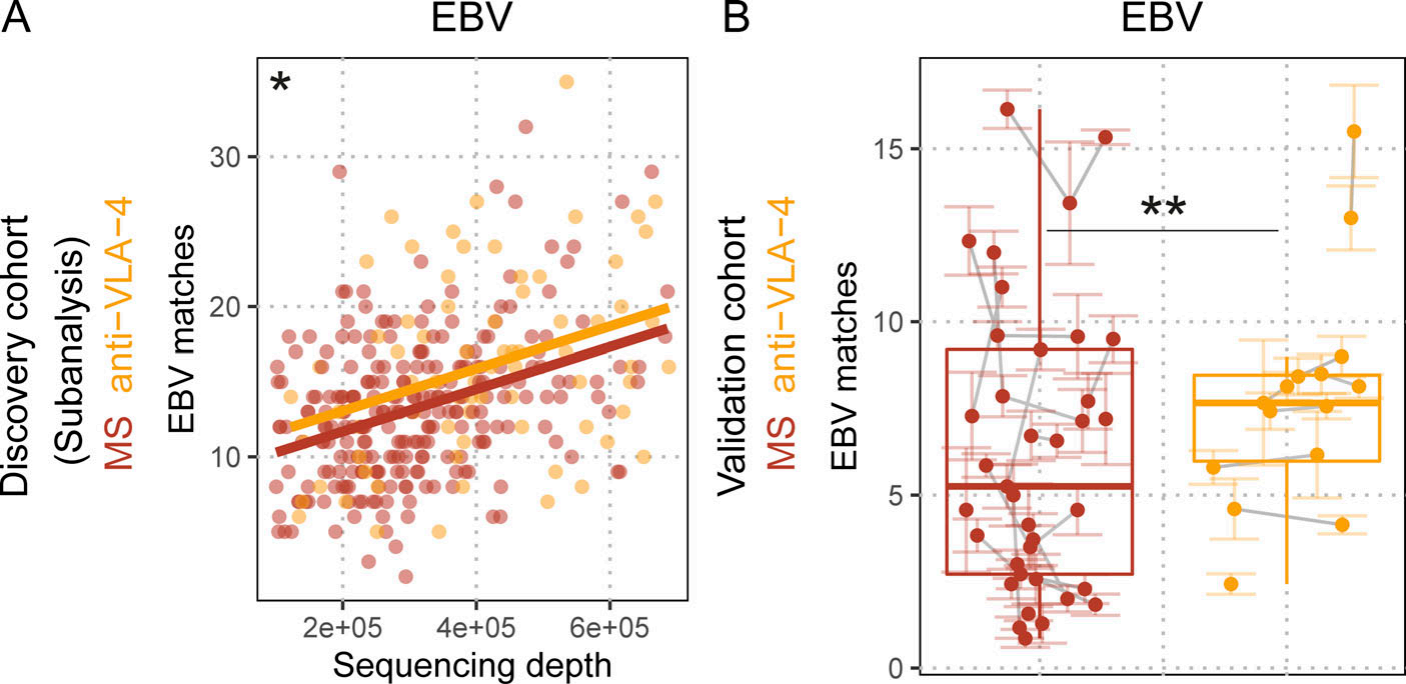
**Treatment with anti–VLA-4 blocking antibody sequesters EBV-specific T cells. (A)** EBV TCRβ sequence matches quantified in untreated MS patients (red dots and line) and anti–VLA-4 blocking antibody-treated MS patients (orange dots and line) against sequencing depth (number of productive templates in the sample; q_anti-VLA-4_ = 0.04156; n_MS_ = 248; n_anti-VLA-4_ = 73); lines indicate linear regressions, *q* values indicate adjusted significance of treatment in linear models with the covariates sequencing depth, age, sex, and HLA. **(B)** EBV TCRβ sequence matches quantified in MS patients (red dots) and anti-VLA–treated MS patients (orange dots; q_anti-VLA-4_ = 0.0081492; n_MS_ = 17; n_anti-VLA-4_ = 8). Colored lines indicate standard error of the mean of the sequencing pools for the respective sample; gray lines connect samples from the same individual. *q* values indicate adjusted significance of anti-VLA–treatment in linear mixed models with the covariates sequencing depth, age, sex, treatment, and sequencing pools nested within samples within individuals.

**Figure S3.**
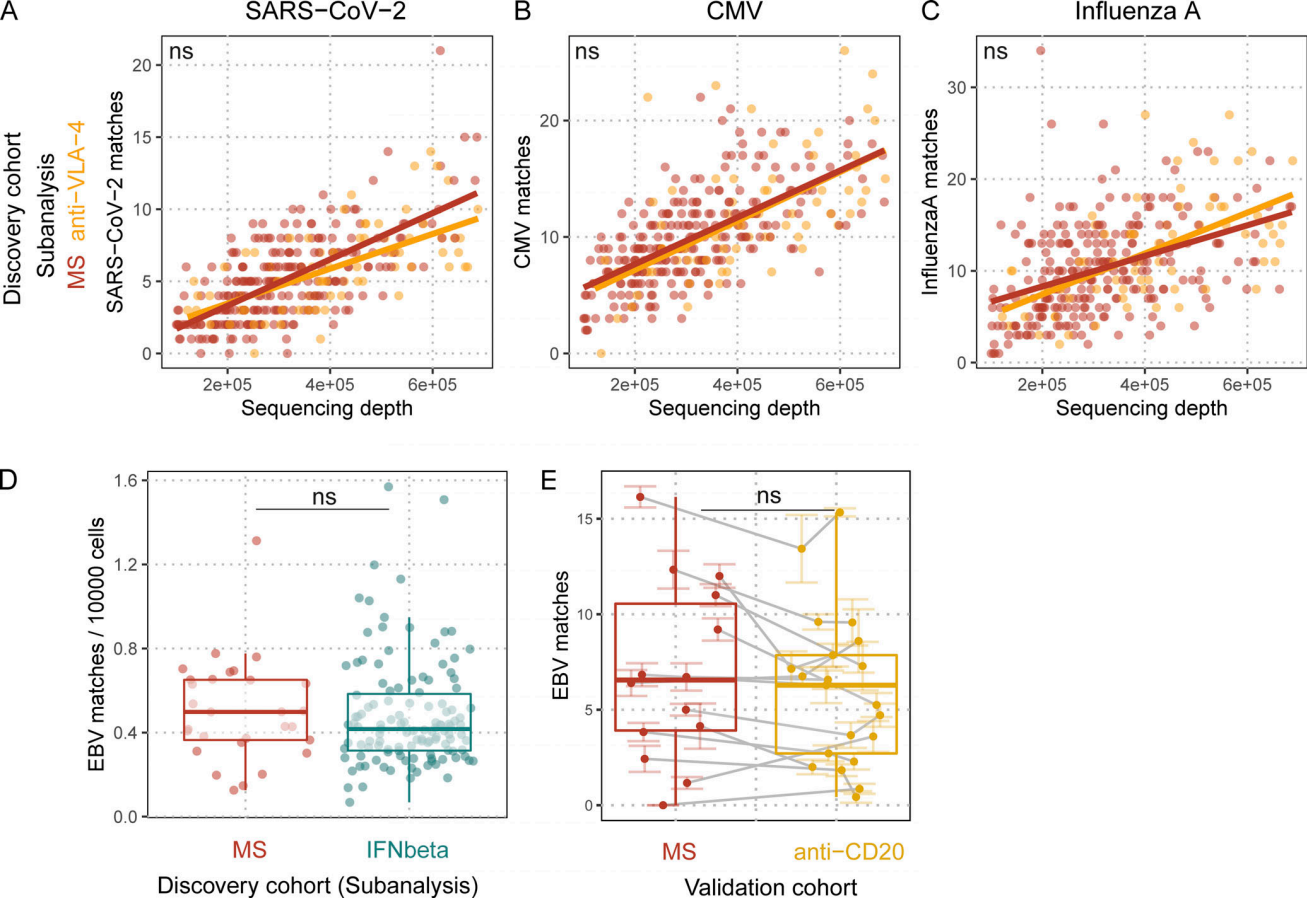
**Quantification of pathogen-specific TCRβ sequences in TCRβ repertoires with regard to MS treatments. (A–C)** SARS-CoV-2 (A), CMV (B), and influenza A (C). TCRβ sequence matches quantified in untreated MS patients (red dots and line) and anti-VLA-4–treated MS patients (orange dots and line) against sequencing depth (number of productive templates in the sample; SARS-CoV-2:q_anti-VLA-4_ = 0.41808; CMV:q_anti-VLA-4_ = 1; influenza A:q_anti-VLA-4_ = 1; n_MS_ = 248; n_anti-VLA-4_ = 73); lines indicate linear regressions; *q* values indicate adjusted significance of treatment in linear models with the covariates sequencing depth, age, sex, and HLA. **(D)** EBV TCRβ sequence matches quantified in treatment-naive MS patients (red dots) and MS patients only treated with IFNβ (cyan dots; q_IFNbeta_ = 1; n_MS_ = 29; n_IFNbeta_ = 123); *q* values indicate adjusted significance of treatment in linear models with the covariates sequencing depth, age, sex, and HLA. **(E)** EBV TCRβ sequence matches quantified in MS patients before their anti-CD20 treatment (red dots), and after their anti-CD20 treatment (yellow dots; q_anti-CD20_ = 0.068; n_MS_ = 14; n_anti-CD20_ = 14). Colored lines indicate standard error of the mean of the sequencing pools for the respective sample; gray lines connect samples from the same individual. *q* values indicate adjusted significance of anti-CD20 treatment in linear mixed models with the covariates sequencing depth, age, sex, treatment, and sequencing pools nested within samples within individuals.

To further evaluate the altered, EBV-specific TCRβ repertoire of MS patients in the context of treatment, we performed a subanalysis on the HLA-A*02–positive donors of the discovery cohort. A subgroup of patients (73 of 321) donated blood alongside treatment with anti–VLA-4, which blocks luminal leukocyte adhesion (Yednock et al., 1995), resulting in peripheral blood sequestration of leukocytes. Therapeutic blockade of immune cell extravasation should not differentiate between antigen specificities if the respective pathogens can be found in these tissues. However, comparison of antigen-specific TCRβ sequences in peripheral blood of treated patients revealed only an increase for EBV-specific sequences (Fig. 3 A; q = 4.2 × 10^−2^), and not any increase for sequences specific for SARS-CoV-2, CMV, or influenza A (Fig. S3, A–C). This finding was also independently validated (Fig. 3 B; q = 8.1 × 10^−3^). As there have been reports of EBV-specific CD8^+^ T cells in the CSF (Bedri et al., 2022; Lossius et al., 2014; van Nierop et al., 2016) and in the brain parenchyma (Serafini et al., 2019) of MS patients, as well as lytic EBV proteins in MS lesions (Moreno et al., 2018), our data would be consistent with an ongoing, potentially compartmentalized immune reaction against EBV in MS patients. This could cause continuous egress of EBV-specific clonotypes from the blood to CNS tissue. EBV reactivation has been shown in a variety of conditions (reviewed by Kerr [2019]) and could contribute to this finding. Blockade of the migratory process could, therefore, specifically accumulate T cells directed against EBV, but not CMV or influenza virus in MS patients. Treatment with IFNβ, which is suspected to elicit antiviral effects, was assessed as a control, but did not change the number of EBV matches (Fig. S3 D). Treatment with anti-CD20 did also not change the number of EBV matches (Fig. S3 E), which suggests that the elevated T cell response might not be dependent on peripheral B cells as an EBV reservoir.

### Immune phenotype of peripheral and CSF EBV-specific CD8 T cells in MS patients and controls

**Figure 4.**
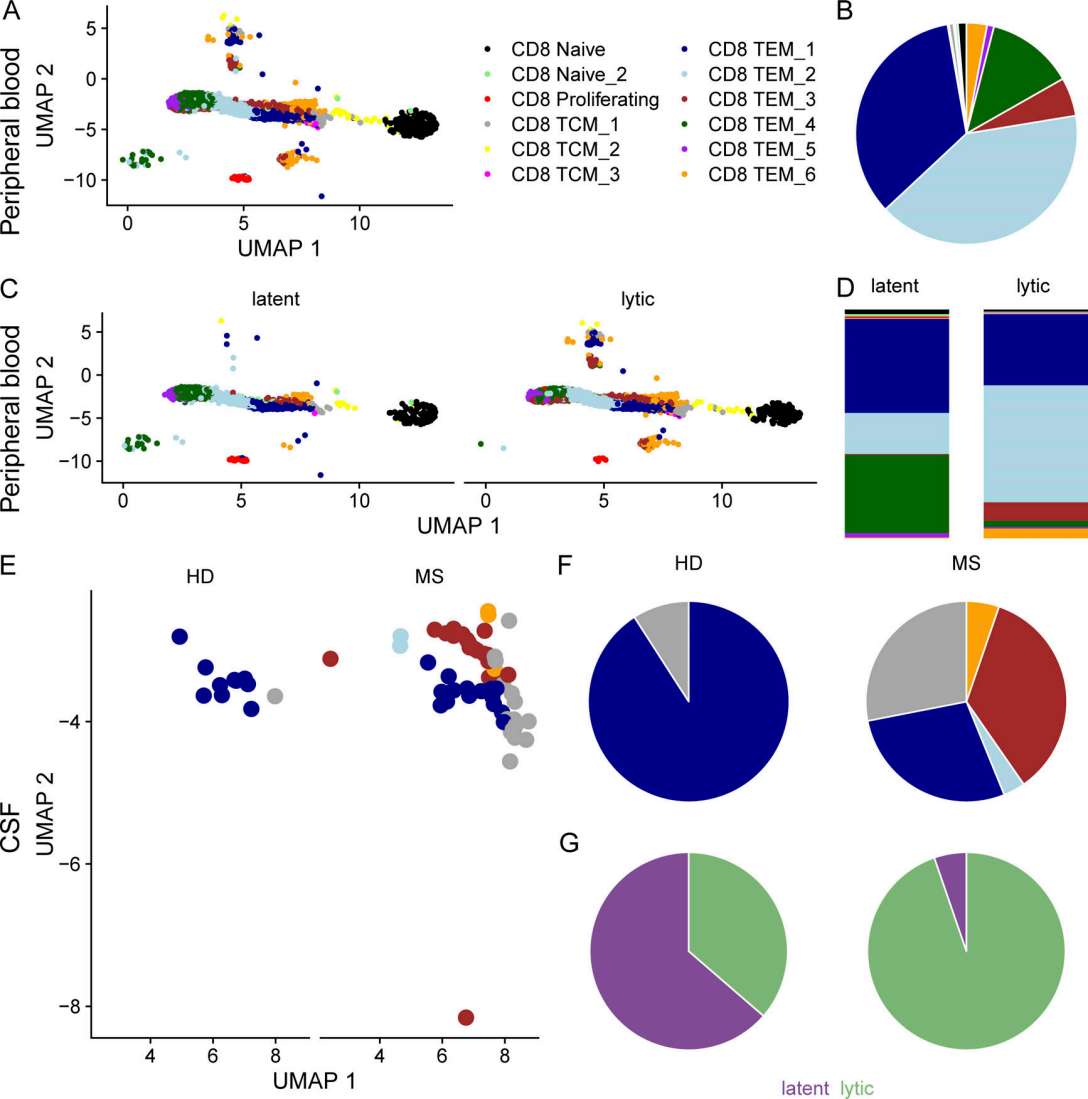
**scRNAseq analysis illustrates phenotype of the EBV-specific CD8**^**+**^ **T cells in HD and MS patients. (A)** UMAP plot of the level-3 granularity mapped CD8^+^ T cells of four deeply sequenced healthy controls previously described by Boutet et al. (2019). Color indicates cluster annotation. **(B)** Quantification of the cluster affiliation of EBV-specific CD8^+^ T cells. **(C)** UMAP plot of the level-3 granularity mapped EBV-specific CD8^+^ T cells, split according to their specificity against latent or lytic EBV epitopes. **(D)** Quantification of latent (left) and lytic (right) cluster affiliation. **(E)** UMAP plot of the level-3 granularity mapped CSF cells from 6 HD and 5 MS patients from Pappalardo et al. (2020). Only EBV sequence–matched CD8^+^ T cells are shown. **(F)** Quantification of the cluster affiliation of EBV-specific CSF cells from HD (left) and MS patients (right). **(G)** Quantification of the specificity against either latent (purple) or lytic (green) proteins of EBV-specific CSF cells from HD (left) and MS patients (right).

To characterize the cellular phenotype of the altered immune response to EBV in MS, the transcriptome of T cells with EBV-matching TCR sequences was assessed and compared between HD and MS patients. To this end, we first analyzed four HD single-cell RNA sequencing (scRNAseq) samples from the public domain, where 128,300 CD8^+^ T cells were sorted from peripheral blood with dextramers specific for 44 different epitopes (Boutet et al., 2019), 12 of them EBV-derived with an overlap of seven epitopes from the database. Most of the CD8^+^ T cells with EBV-matching TCR sequences were located in two subsets of the effector-memory T cell (TEM) compartment, characterized by gene signatures of *PDCD1*, *CD28*, and *KLRK1*/*NKG2D* for TEM_1 and *TIGIT*, *NCAM1*, and *CD244* for TEM_2 (Hao et al., 2021; Fig. 4, A and B). Matching cells were then divided by specificity against either latent or lytic EBV epitopes, which showed that the latent response consisted of a larger number of TEM_4 cells (characterized by *NGFR*, *SPN*, and *ICOSLG*), but fewer TEM_2 cells (Fig. 4, C and D). To assess, whether the EBV response is altered in MS patients, CSF scRNAseq samples of six HD and five MS patients (Pappalardo et al., 2020) were analyzed and the EBV-specific matches were annotated. MS patients presented with more EBV matches in the CSF, which has been suggested before (Bedri et al., 2022; Lossius et al., 2014; van Nierop et al., 2016), and more interestingly, these sequence matches were predominantly directed against lytic epitopes and also differed in phenotype: HD CSF contained almost exclusively EBV-specific TEM_1 with 36% of sequences specific for lytic epitopes, whereas in MS CSF, the EBV-specific CD8^+^ T cells distributed evenly between TEM_1, TEM_3, and TCM_1 with 95% of sequences specific for lytic epitopes. TEM_3 cells are characterized by *ITGA4* and *ITGB1* (α and β chains of VLA-4), and *CCR5*, while TCM_1 being characterized by *ITGAE*, *KLRK1*, and integrin β 7 expression (*ITGB7*). As central-memory T cells in CSF are generally thought to be part of MS pathology (Kivisakk et al., 2004), have recently been primed in contrast to effector-memory T cells (Jameson and Masopust, 2018), and have specifically been shown to be required for a protective lytic EBV response (Deng et al., 2021), the results would be consistent with a recent antigen exposure of TCM in an ongoing immune reaction against EBV. Additionally, CD103 surface expression (encoded by *ITGAE*) is considered a marker for tissue-resident memory cells (Kumar et al., 2017), which have been identified as part of CNS pathology in MS (Fransen et al., 2020; Machado-Santos et al., 2018).

### Concluding remarks

Caveats of this study are that only the TCRβ chain was assessed, whereas true determination of specificity can only be achieved with paired TCR α/β chains. However, as the analysis yielded significant results in the anti–SARS-CoV-2 response of COVID-19 patients and in vaccinated HD, in the anti-CMV response in CMV seropositive individuals, and the finding pertaining to EBV and MS could be shown in four independent cohorts, we are confident that these results would only get stronger in a paired analysis. Additionally, all 528 database-derived, multimer-confirmed EBV-specific sequences were MHC-I restricted. Therefore, B cell help by CD4^+^ T cells especially in the context of the MS risk allele DRB1*15 could not be evaluated. Lastly, quantifying EBV-specific TCRβ sequences does not allow for a judgment on the quality of the T cell response to EBV.

Taken together, our study suggests that MS is not only associated with a higher EBV antibody seroprevalence (Bjornevik et al., 2022), but also with a broader EBV-specific TCR repertoire, even in monozygotic twin pairs with matching, seropositive EBV status, and identical genetics, as well as early childhood environment. Future studies should determine whether this broader repertoire against EBV in MS is just a byproduct of a putatively aberrant, immune response, or involved in driving disease pathology.

## Materials and methods

### Sequencing

High-throughput ultradeep resolution TCRβ chain sequencing of genomic DNA from unsorted peripheral blood was performed with the immunoSEQ Assay in collaboration with Adaptive Biotechnologies (Carlson et al., 2013; Robins et al., 2009), or with the immunoPETE assay in collaboration with Roche (Dannebaum et al., 2022). Three cohorts were sequenced, the discovery cohort and MS twin cohort with immunoSeq, the validation cohort with immunoPETE (Table 1). In the case of the immunoPETE assay (Dannebaum et al., 2022), several time points/samples were sequenced from most individuals and each sample was split into five to seven parts, which we consider biological replicates of a sample. The resulting five to seven replicates were sequenced separately to be able to account for possible batch effects. Missing HLA information for the COVID-19 patients and the validation cohort was imputed according to previously published methods (Emerson et al., 2017).

### Cohorts

Detailed cohort characteristics are listed in Table 1. The discovery cohort encompassed 1336 MS patients (1241 RRMS and 95 clinically isolated syndrome patients), described in Cohen et al. (2021); Gross et al. (2021); Outteryck et al. (2014) and 229 HD, as well as 607 COVID-19 patients as positive control (Snyder et al., 2020). The MS twin cohort encompassed 35 monozygotic twin pairs discordant for MS (Gerdes et al., 2020; Ingelfinger et al., 2022). The validation cohort encompassed 59 RRMS patients, 11 HD, and 40 autoimmune encephalitis patients, added in as non-MS inflammatory controls. Informed written consent was obtained from all participants and the study was performed according to the Declaration of Helsinki. While the serological EBV status of the controls from the discovery cohort was not available, the mean age of 37 (MS) and 55 (HD) would suggest that all individuals had a seropositive EBV status (Abrahamyan et al., 2020). In the MS twin cohort, all individuals were serotyped as being EBV positive.

### TCR repertoire analysis

TCRβ repertoires were queried against public databases (download in April 2022) of HLA-restricted TCRβ rearrangements specific for four common pathogens from the VDJdb (SARS-CoV-2, EBV, CMV, and influenza A virus; database confidence score >0; Bagaev et al., 2020) plus SARS-CoV-2–specific rearrangements from the IEDB (Vita et al., 2019) in order to have a comparable number of sequences in each category (644 SARS-CoV-2, 528 EBV, 840 CMV, 381 influenza A). Database TCRβ sequences with a CDR3 amino acid length smaller than six, or with missing information (organ, protein, epitope, HLA restriction, variable β chain family) were excluded (Table S1). HLA-A*02–positive individuals were preselected due to the known reduced allelic frequency of HLA-A*02 in MS patients (International Multiple Sclerosis Genetics consortium et al., 2011) and to maximize detection power, because most published sequences in the database were restricted to this allele. Preselection for the MS risk allele HLA-DRB1*15 was not necessary, as there were no sequences for the four chosen pathogens restricted to this allele. Twins were not preselected, as there is no HLA bias in a paired monozygotic twin sibling analysis. Subsequently, all TCR sequences were collected for each individual from the databases restricted to the individual’s HLA alleles and matched using the TCR Vβ family and the amino acid sequence of the CDR3. Matches were then aggregated according to the pathogen. In case of the epitope subanalysis, only TCR sequences for a specific epitope were matched in individuals positive for the HLA allele for which the epitope-specific multimer was restricted.

### scRNAseq analysis

The following datasets were used for the scRNAseq analysis: four HD, where cells were labelled with 44 different epitope/HLA specific dextramers and then sorted and subsequently sequenced using 10x Genomics protocols (Boutet et al., 2019; Zenodo accession number 6952657), as well as CSF data from six HD and five MS patients from Pappalardo et al. (2020). The data from Pappalardo et al. (2020) were financially supported by the National Institutes of Health and downloaded from the National Institutes of Health’s dbGaP database (accession phs002222.v2.p1). Data were combined into Seurat objects, SCtransformed, and mapped according to the level 1, 2, and 3 granularities provided by the Seurat package (Hao et al., 2021). Cells were then subset to CD8^+^ T cells (level 1) and the CD8^+^ T cell subclusters of level 2, before the level 3-granularity cluster distribution was analyzed. Matching of the TCR sequences with the database entries was performed using the R package *stringdist* (van der Loo, 2014) with a Levenshtein distance of ⩽1 defining a match.

### Statistical analysis

Significance was assessed using linear models for the cross-sectional analysis and linear mixed models for the twin and validation cohort. The former included the covariates sequencing depth, age, sex, and disease status (MS, yes/no; and COVID-19, yes/no), the latter included sequencing depth, age, MS status, and twinship (Gerdes et al., 2020). In case of the MS twin study, there was no need to adjust for sex, but as the blood samples were not always drawn on the same day, the model had to include age. Additionally, the MS twin study models included information about symptomatic EBV infection in childhood/infectious mononucleosis. HLA information for 16 restriction alleles from the TCRβ sequence databases was added to the models of the discovery and MS twin cohort, but not the validation cohort, due to the lower number of patients. False discovery rate adjustment of model P values for multiple comparisons with the number of assessed pathogens (4) resulted in the displayed *q* values (Bonferroni, 1936). The immunoPETE samples were analyzed in a nested model (pools within samples within individuals) and adjusted for sequencing depth (number of input α β T cells), age, sex, MS, vaccination, and anti–VLA-4/anti-CD20 treatment. The epitope subanalysis models did not include the HLA information, as the donors and sequences were all matched/restricted to one epitope-specific HLA allele. The lasso regression models to evaluate MS association of EBV-specific sequences in the HLA-A*02–positive individuals of the discovery cohort were performed using the *glmnet* R package (Simon et al., 2011) with the factor MS as target variable, the individuals’ HLA alleles as the covariates, an α value of 0 and 3 folds.

### Online supplemental material

Table S1 lists the pathogen-specific TCRβ sequences from the databases VDJdb and IEDB queried for this study, including organism, protein, epitope, and HLA information, as well as the sequence matches of the discovery and MS twin cohort for each individual. Table S2 details the numbers of EBV-specific TCR sequences found in HLA classifier patterns. Table S3 details the model parameters of the HLA-restricted, EBV epitope–specific subanalyses of the discovery, and MS twin cohort, including sequence and donor *n* numbers, estimates, errors, and P values. Fig. S1 depicts quantification of pathogen-specific TCRβ sequences in TCRβ repertoires. Fig. S2 defines a minimal set of MS-associated EBV-specific TCRβ sequences and depicts their quantification in the validation cohort. Fig. S3 depicts quantification of pathogen-specific TCRβ sequences in TCRβ repertoires with regard to MS treatments.

## Supplementary Material

Table S1 TCRβ sequences from the databases VDJdb and IEDB queried in this study.

Table S2 Number of EBV-specific TCR sequences found in HLA classifier patterns.

Table S3 Model parameters of the HLA-restricted epitope-specific subanalyses. Shown are the EBV protein, EBV epitope, EBV virus cycle of the protein, MHC restriction element of the multimer staining that resulted in the TCR sequences, number of available TCR sequences, number of HD, MS patients, and COVID-19 patients for that analysis, the model estimate (effect size), standard error, *t* value, and P value of the model, as well as the cohort in which the model was run.
