# Correction: Broader Epstein–Barr virus–specific T cell receptor repertoire in patients with multiple sclerosis

**DOI:** 10.1084/jem.2022065010252022c

**Published:** 2022-10-28

**Authors:** Tilman Schneider-Hohendorf, Lisa Ann Gerdes, Béatrice Pignolet, Rachel Gittelman, Patrick Ostkamp, Florian Rubelt, Catarina Raposo, Björn Tackenberg, Marianne Riepenhausen, Claudia Janoschka, Christian Wünsch, Florence Bucciarelli, Andrea Flierl-Hecht, Eduardo Beltrán, Tania Kümpfel, Katja Anslinger, Catharina C. Gross, Heidi Chapman, Ian Kaplan, David Brassat, Hartmut Wekerle, Martin Kerschensteiner, Luisa Klotz, Jan D. Lünemann, Reinhard Hohlfeld, Roland Liblau, Heinz Wiendl, Nicholas Schwab

Vol. 219, No. 11 | https://doi.org/10.1084/jem.20220650 | September 1, 2022

During data analysis for a follow-up project, the authors found that the number of patients who received ocrelizumab infusions in the metadata underlying Fig. S3 E was incorrect. The corrected Fig. S3 with the revised panel E is provided here, and the legend has been changed as indicated in bold. In addition, in Table 1, the validation cohort data in the “Anti-CD20: before/after treatment” row now read “14/14” instead of “25/17” (shown in red text here). The conclusions regarding these data are unchanged. The errors appear in PDFs downloaded before October 25, 2022.

**Figure S3.**
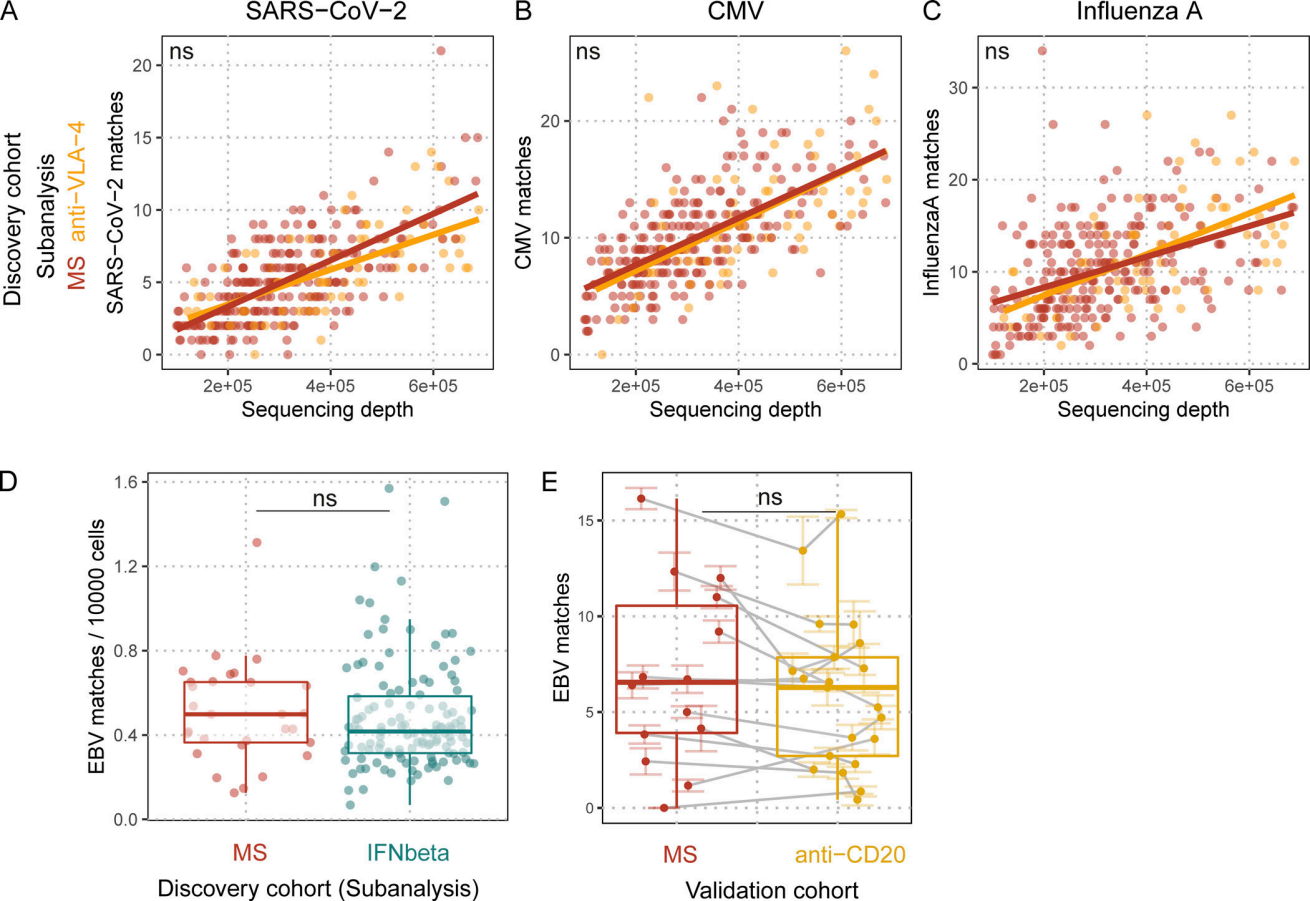
Quantification of pathogen-specific TCRβ sequences in TCRβ repertoires with regard to MS treatments. **(A–C)** SARS-CoV-2 (A), CMV (B), and influenza A (C). TCRβ sequence matches quantified in untreated MS patients (red dots and line) and anti-VLA-4–treated MS patients (orange dots and line) against sequencing depth (number of productive templates in the sample; SARS-CoV-2:q_anti-VLA-4_ = 0.41808; CMV:q_anti-VLA-4_ = 1; influenza A:q_anti-VLA-4_ = 1; n_MS_ = 248; n_anti-VLA-4_ = 73); lines indicate linear regressions; q values indicate adjusted significance of treatment in linear models with the covariates sequencing depth, age, sex, and HLA. **(D)** EBV TCRβ sequence matches quantified in treatment-naive MS patients (red dots) and MS patients only treated with IFNβ (cyan dots; q_IFNbeta_ = 1; n_MS_ = 29; n_IFNbeta_ = 123); q values indicate adjusted significance of treatment in linear models with the covariates sequencing depth, age, sex, and HLA. **(E)** EBV TCRβ sequence matches quantified in MS patients before their anti-CD20 treatment (red dots), and after their anti-CD20 treatment (yellow dots; **q**_**anti-CD20**_ **= 0.068; n**_**MS**_ **= 14; n**_**anti-CD20**_ **= 14**). Colored lines indicate standard error of the mean of the sequencing pools for the respective sample; gray lines connect samples from the same individual. q values indicate adjusted significance of anti-CD20 treatment in linear mixed models with the covariates sequencing depth, age, sex, treatment, and sequencing pools nested within samples within individuals.

**Table 1.**
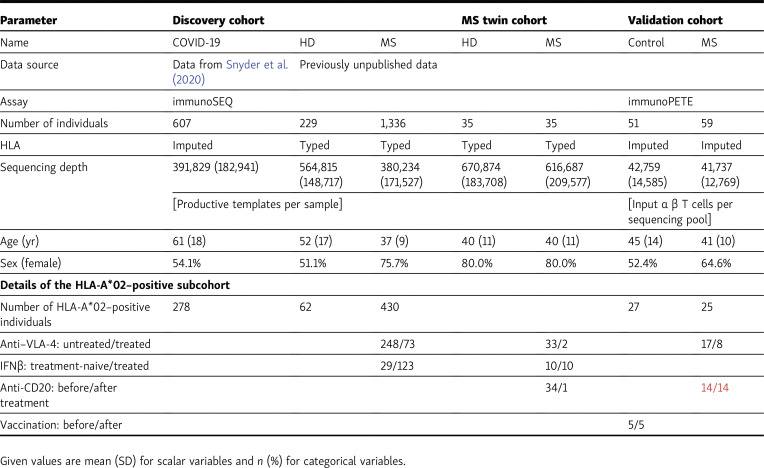
Cohorts and sequencing characteristics

